# How to Study Drugs

**Published:** 1887-08

**Authors:** 


### HOMŒOPATHIC PHYSICIAN,

A MONTHLY JOURNAL OF MEDICAL SCIENCE.

"If our school ever gives up the strict inductive method of Hahnemann, we are lost, and deserve only to be mentioned as a caricature in the history of medicine."—CONSTANTINE HERING.

Vol. VII. AUGUST, 1887. No. 8.

### EDITORIAL NOTES.

HOW TO STUDY DRUGS.—In a recent issue of a journal we read that some physicians, wishing to ascertain the probable value of Cimicifuga in parturition, gave it in frequent doses to every patient that came to the Maternity Hospital. The results as given were of course unsatisfactory; they could only be compared with usual unassisted labors. The object in this trial was to ascertain if Cimicifuga gave beneficial assistance to the parturient female. The attempt was made to ascertain this by the above-mentioned method of giving the drug. In some of the patients the labor was easier, in others about the average, and in some, maybe, more difficult than usual. Hence the net results were about the same as if no medicine had been used.

Our purpose in mentioning this testing of Cimicifuga is to call attention to the method followed. It is an old one, and one which has never yielded any beneficial results. It is a faulty method, for it presumes that all women are alike in their constitutions and in their symptoms. It has been by following some such method as this that the allopaths have built their system of fallacious therapeutics. It has been followed by continued changing of theories and of drugs. To-day we hear of a medicine having been used in such and such a disease with wonderful results. It is said that at last a cure for some dangerous disease has been found, and great boasting is made over this discovery. Very soon we are told that this medicine has disappointed its advocates. The story is an old one; it need not be enlarged upon.

In contrast to this method of studying drugs we have the system adopted by Hahnemann—a system which has produced such beneficial results that it has been slyly adopted by the allopaths. Hahnemann contended that a drug would cure in the sick such symptoms as it produced upon the healthy. Here was the radical change, testing drugs upon the healthy, not upon the sick. By this plan of studying drug action we are enabled to ascertain with certainty the sphere of usefulness of each drug. By its teaching we know that no one drug can aid all cases of any disease. By this method of studying drugs we know that neither Ergot nor Cimicifuga will aid every case of parturition. We know that each of these drugs has its sphere, in which it will always do good work; we know, also, that we are not limited to these two drugs, but that any drug may be needed in a case of parturition, and that one drug will be as effective as another when properly prescribed.

Hahnemann's method has not only given us certain knowledge of drugs, it has also given us many drugs for each disease.

